# Reduced graphene oxide composites with water soluble copolymers having tailored lower critical solution temperatures and unique tube-like structure

**DOI:** 10.1038/srep44508

**Published:** 2017-03-14

**Authors:** Mina Namvari, Chandra S. Biswas, Massimiliano Galluzzi, Qiao Wang, Bing Du, Florian J. Stadler

**Affiliations:** 1 College of Materials Science and Engineering, Shenzhen Key Laboratory of Polymer Science and Technology, Guangdong Research Center for Interfacial Engineering of Functional Materials, Nanshan District Key Lab for Biopolymers and Safety Evaluation, Shenzhen University, Shenzhen 518060, PR China; 2 Key Laboratory of Optoelectronic Devices and System of Ministry of Education and Guangdong Province, College of Optoelectronic Engineering, Shenzhen University, Shenzhen, People’s Republic of China

## Abstract

Nanohybrids of graphene with water soluble polymer were synthesized using ‘grafting from’ method. GO, prepared by modified Hummers’ method, was first reacted with sodium azide. Alkyne-terminated RAFT-CTA was synthesized by reaction of propargyl alcohol and *S*-1-dodecyl-*S’*-(α,α‘-dimethyl-α”-acetic acid) trithiocarbonate. RAFT-CTA was grafted onto the GO sheets by facile click-reaction and subsequently, *N*-isopropylacrylamide (NIPAM) and *N*-ethyleacrylamide (NEAM) were polymerized on graphene sheets via RAFT polymerization method. The respective copolymers with different ratios were also prepared. The nanohybrids were characterized by FTIR, XRD, TGA, Raman, SEM, and AFM. Both SEM and AFM clearly showed rod-like structures for rGO-PNEAM. XRD showed a small peak at 2θ = 19.21°, corresponding to d-spacing ≈ 4.6 Å. In addition, the nanohybrids showed a very broad temperature range for the LCST in water between ca. 30 and 70 °C.

Polymer nanocomposites based on carbonaceous materials such as carbon black, carbon nanotubes (CNTs), or fullerenes are used to improve the mechanical, thermal, electrical, and gas barrier properties of polymers^1^,^2^,^3^,^4^. The discovery of graphene^5^ with its combination of extraordinary physical properties and ability to be dispersed in various polymer matrices has created a new class of polymer nanocomposites^6^,^7^. Graphene is hydrophobic, thus, producing water-dispersible graphene nanocomposites has always been of great interest, which can be achieved by modification with water-soluble polymers. The high density of oxygen functionalities on the surface of basal planes of graphene oxide (GO) and at the sheet edges, arising from oxidation of natural graphite powder with various oxidants in acidic media, offers the potential for solution processing and further functionalization, and makes GO an important precursor of graphene sheets^8^. Polymer materials have been covalently ‘grafted from’ or ‘grafted to’ GO-nanosheets, through oxygen-containing functional groups, via surface-initiated polymerization^9^,^10^,^11^,^12^, esterification^13^,^14^, amidation^15^,^16^, click-chemistry^17^,^18^,^19^,^20^,^21^,^22^, and isocyanate modification^23^. The ‘grafting to’ approach is most commonly used methods for tethering polymers onto GO-nanosheets, in which polymer chains are attached directly to oxygen functionalities or other previously-attached surface molecules or at the GO-edges through simple chemical reactions^24^,^25^. However, this approach is limited by steric hindrance of the polymer chains. The alternative ‘grafting from’ approach, or surface-initiated polymerization from pre-immobilized initiators, is capable of achieving a higher grafting density^26^. Graphene and GO have been modified with various polymers such as polystyrene^27^,^28^,^29^, polyaniline^30^, poly(2-hydroxyethyl acrylate)^9^, poly(N-vinyl pyrrolidone)^31^, and poly(N-isopropylacrylamide)^11^ using the ‘grafting from’ method.

Click-reactions^32^ are powerful tools to connect different molecules^33^,^34^,^35^ such as attaching reversible addition-fragmentation chain transfer (RAFT) agents^11^, nanoparticles^36^, and carbohydrates^37^ onto GO. We have reported carbohydrate and magnetic nanoparticles attachment onto GO via click-reaction^33^,^35^. To our knowledge, there are not many reports on using ‘grafting from’ method, RAFT-polymerization and click reaction together to prepare graphene nanocomposites.

Since graphene has very high electrical conductivity and very high stiffness, polymer-graphene nanohybrids demonstrate improved mechanical and electrical properties. Hence, graphene nanocomposites are useful as sensing materials due to high specific surface area and electrical properties of graphene^38^. However, oxygen-containing functional groups and the covalently attached polymer chains may disrupt the graphitic π-conjugation network, with severe scattering of ballistic charge transport^24^. As a result, GO–polymer nanosheets prepared by covalent functionalization are usually insulating in nature^39^. Thus, reduction of GO sheets after functionalization is essential to obtain sufficiently conductive materials. Ascorbic acid, which reduces Cu^2+^ to Cu^1+^ during click reaction, is also a green reducing agent for GO^40^. Hence, reduction happens during click reaction.

In this paper, we report modification of GO with RAFT-CTA using click reaction followed polymerization of *N*-isopropylacrilamide, *N*-ethylacrylamide, and their copolymers. The aforementioned monomers were polymerized on graphene sheets via RAFT-polymerization method. By using the covalent functionalization strategy, stable aqueous dispersions of rGO–polymer nanosheets with adequately controlled nanostructures and enhanced compatibility with the polymer matrices were obtained.

## Results and Discussion

**Figure 1:**
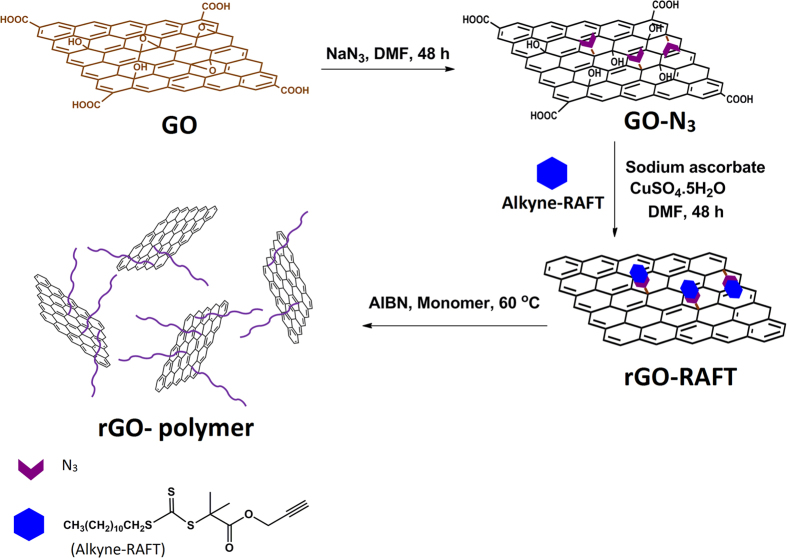
Preparation of rGO-polymer nanohybrids.

**Figure 2:**
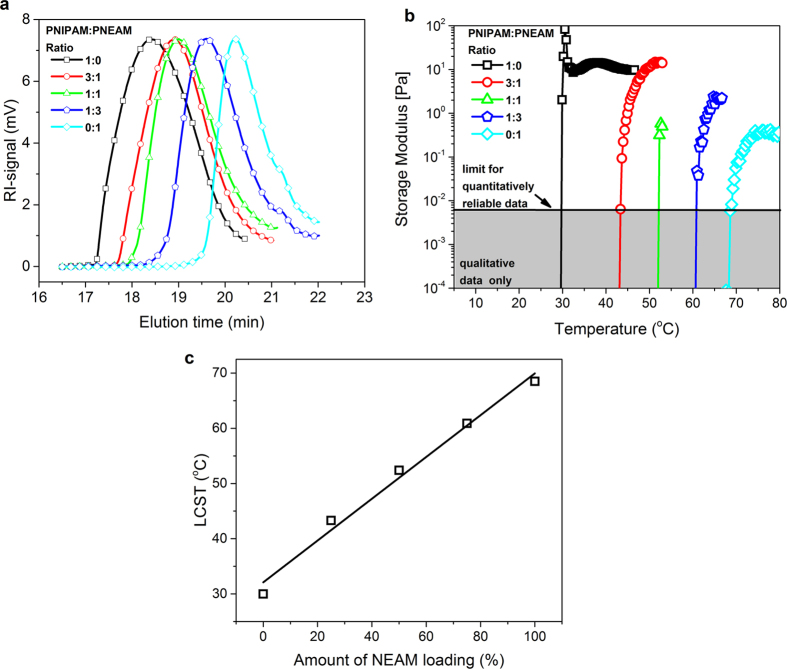
(**a**) GPC-data of sacrificial polymers, (**b**) storage moduli as a function of temperature, (**c**) LCSTs deducted from (**b**).

The synthetic procedure is shown in Fig. 1. FTIR (Fig. SI1) and Raman (Fig. SI2) spectra confirm the completion of every step. We used the free polymers for SEC, to determine the number average molar mass M_n_ of the grafted polymer in nanocomposites (Fig. 2). According to the mechanism of living radical polymerization, those possess almost same molar mass as grafted ones^41^. The highest average molar mass *M*_n_ was observed for PNIPAM. *M*_n_ decreased with increasing PNEAM concentration. As PNEAM is more hydrophilic than PNIPAM, driving forces for detaching RAFT-agents from hydrophobic rGO-RAFT upon polymerization with NEAM are superior to PNIPAM. The *M*_n_-value and PDI observed for PNEAM were 1000 g/mol and 1.34 and for PNIPAM 6800 g/mol and 1.31, respectively. The copolymers’ molar masses varied very systematically between both homopolymers (Table 1). This system introduced us a good prospect for not only tuning the effective chain length over a wide range, but also gave a very good opportunity of tuning the hydrophilicity of the grafted polymer chains.

The determination of LCSTs of the polymer nanocomposites were done by rheological measurements. Aqueous solutions of the sacrificial polymers (10%(w/v)) were prepared in water and temperature sweep measurements (10 °C–90 °C). It is evident that the LCST of the polymers gradually increased with increasing NEAM loading (Fig. 2b). For PNIPAM, the expected LCST around 32 °C was found, while PNEAM’s LCST was found to be between 68 and 82 °C^42^,^43^. The copolymers’ LCST varied systematically monomer composition dependent and almost linearly between these two (Fig. 2c).

LCST is a thermodynamic phenomenon and observed at the temperature when a polymer turned from coil-to-globular state due the release of water from the polymer matrix owing to increasing intra-molecular hydrophilic interactions among amide groups and hydrophobic interactions among polymer backbone and side-chain alkyl groups. Here, depending on the hydrophilic nature of the polymers, LCST varied almost linearly. A linear dependence of LCST with monomer compositions indicated that the monomers were statistically inserted during polymerization, which is confirmed by absence of broadening of storage modulus increase for the copolymers, as a broadening (or in case of a diblock copolymer a double transition) would indicate a significant deviation from statistical insertion. So, formation of polymer nanocomposites of different hydrophilicity and, thus, LCST is possible by solely varying monomer ratios.

**Figure 3:**
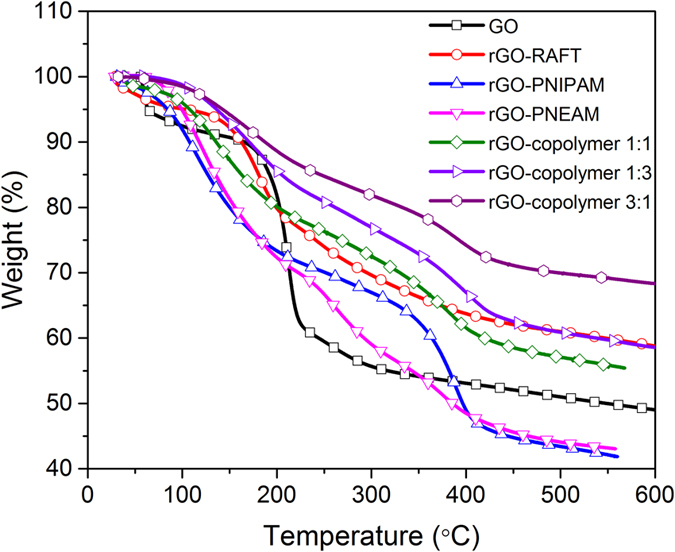
TGA of GO, rGO-RAFT, and polymer nanohybrids.

Figure 3 shows the TGA of GO, rGO, and nanohybrids. TGA curve of GO shows a loss of adsorbed water below 100 °C. The main decompositions occurred around 200 °C. This were mainly due to the decomposition of labile epoxy, hydroxyl, and carboxylic groups. The final mass remained approximately constant at T > 550 °C at ca. 50%. This was close to the previously reported values. The rGO-RAFT followed almost similar trend but final mass was considerably higher (~60%) than GO. The reason for this was the oxygen containing groups were mostly absent here which reflected in less mass loss than GO. Upon grafting with PNIPAM and PNEAM the mass loss process also started below 200 °C, mainly due to the remaining labile oxygen containing groups. However, the onset of TGA curve of the rGO-polymer nanocomposites were lower and less stable compared to rGO-RAFT. This is due to the reduced van der Waals interactions between graphene layers owing to the introduction of polymers in between the sheets. The major mass losses occurred just below 400 °C due to the decomposition of the polymer chains. Their final masses were close to each other (just above 40%) but much less than rGO-RAFT. On the other hand, the rGO-copolymer nanocomposites followed almost similar trend like the rGO-homopolymer nanocomposites but the final mass was significantly higher than homopolymer nanocomposites and slightly less than rGO-RAFT (~55%). This result suggested that the grafting rate of the random copolymer on rGO-RAFT surface has decreased with the increase in the hydrophilicity of the monomer. This is mainly due to the highly hydrophobic nature of reduced graphene oxide which is reluctant to grafting highly hydrophilic polymer on to it.

**Figure 4:**
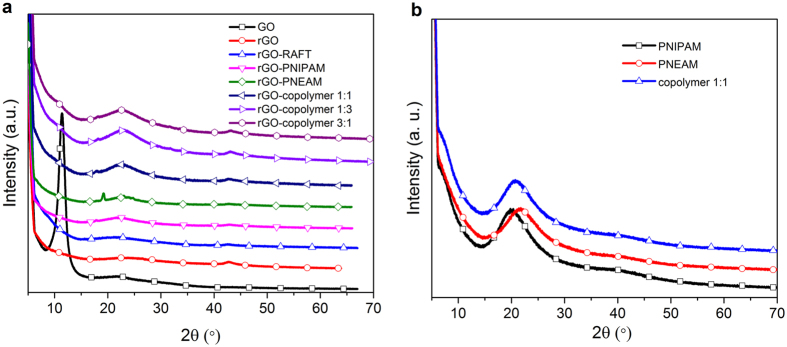
(**a**) XRD patterns of GO, rGO-RAFT, and polymer nanohybrids and (**b**) TGA curves of GO, rGO-RAFT, and polymer nanohybrids.

XRD patterns of graphene nanohybrids are shown in Fig. 4a. The (002) peak of graphite (not shown here) shifted to 11.46° in GO after oxidation and a clear change in intensity and broadness was observed^44^. After functionalization with RAFT-CTA, this peak shifted back to its original (002) peak at 22.20°, confirming the reaction between GO-N_3_ and RAFT-CTA and organic molecule intercalation into the interlayer spacing of GO. Moreover, it showed a pattern similar to rGO that could also indicate the reduction of GO with sodium ascorbate during click reaction and partially restoring of its electronic conjugation^36^. Polymer nanohybrids showed broad peaks at 22.20° and 22.13° for rGO-PNIPAM and rGO-PNEAM, respectively, while the sacrificial polymers showed broad peaks with higher intensity at 20.02° and 21.56 (Fig. 3b). For rGO-PNEAM, a sharp peak with low intensity was observed at 19.21°, indicating some kind of crystalline structure to be discussed later.

**Figure 5:**
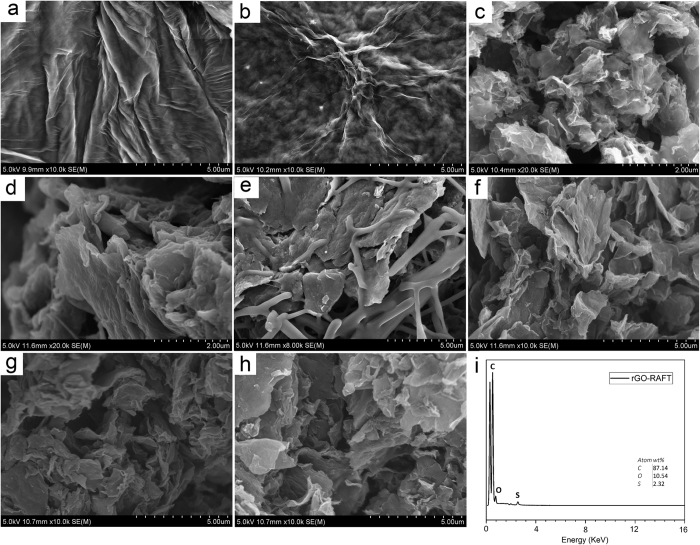
SEM images of (**a**) GO, (**b**) GO-N_3_, (**c**), rGO-RAFT, (**d**) rGO-PNIPAM, (**e**) rGO-PNEAM, and (**f**) rGO copolymer 1:1, (**g**) rGO copolymer 1:3, (**h**) rGO copolymer 3:1, and (**i**) EDX of rGO-RAFT.

SEM images are shown in Fig. 5. Large wrinkled sheets of GO were clearly observed in Fig. 5a, while the surface got rougher after functionalization (b, c) and also smaller in case of rGO-RAFT, which was also confirmed by AFM data. This roughening can be easily understood, when considering that 0.24 mmol g^−1^ RAFT-agents grafted onto the sheets mean that the typical distance between 2 graft sites is almost twice as long as the stretched length of the RAFT-agent including the connecting group. Hence, the surface of the rGO-RAFT must be rougher as the RAFT-agents cannot form a close-packed layer for geometrical reasons. The exact reasoning for these geometrical calculations is given in SI. Polymerization has led to even rougher surface and in case of rGO-PNEAM some tubular structures were observed which are confirmed by AFM data. EDX data of rGO-RAFT also confirmed the reduction during click-coupling reaction (Fig. 5i). TEM images are shown in Fig. SI4.

**Figure 6:**
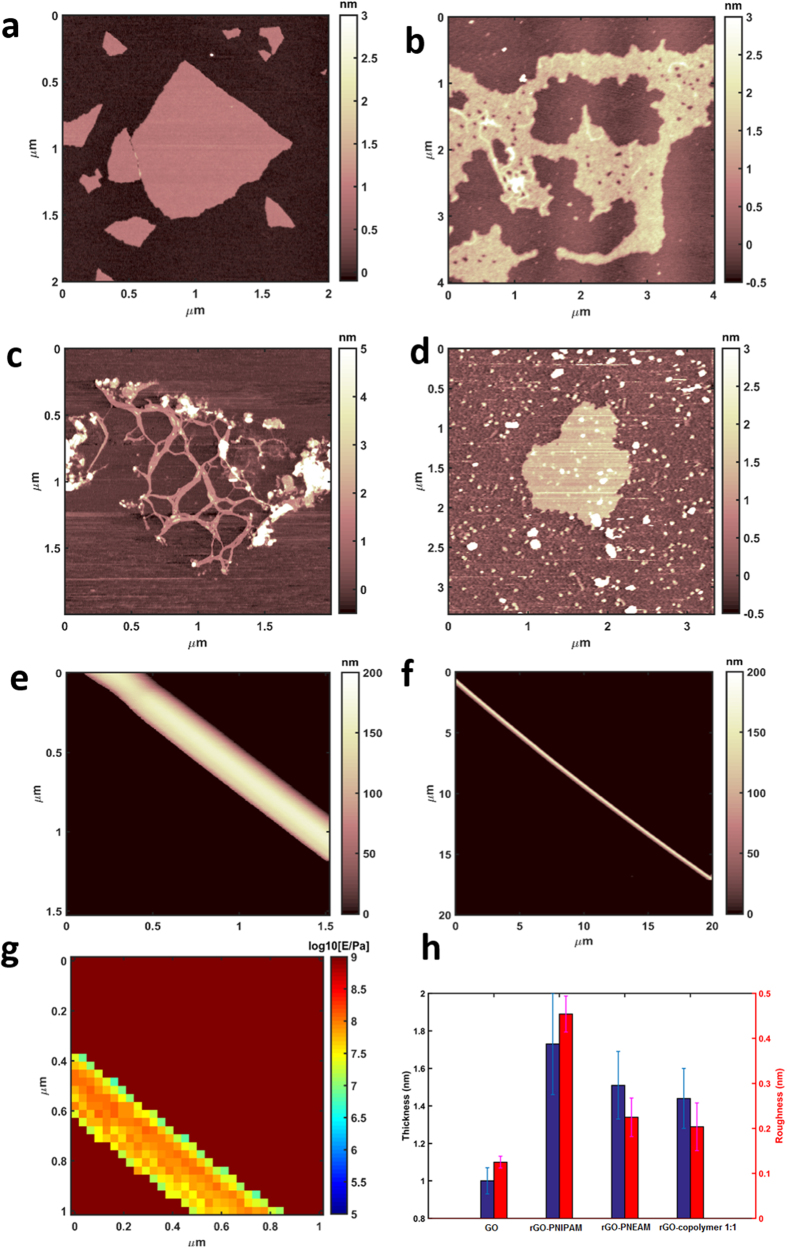
AFM images of (**a**) GO, (**b**) rGO-PNIPAM, (**c**) rGO-PNEAM, (**d**) rGO-copolymer 1:1, (**e,f**) tubular structure in rGO-PNEAM, (**g**) mechanical spectroscopy, (**h**) thickness and roughness of GO and polymer composites.

As observed in SEM-images of rGO-PNEAM, long and straight tubular structures were seen in AFM-images (Fig. 6c), while rGO-PNIPAM did not(Fig. 6b). Mechanical spectroscopy revealed a structure sensitive to indentation (Young’s modulus ~80 MPa, Fig. 6g), suggesting hollow tubes, which speculatively were rolled up graphene sheets, which resulted in the sharp XRD-peak for rGO-PNEAM at 2θ = 19.21° (Fig. 4a).

This conclusion is analogous to multi-walled carbon nanotubes (MWCNT), which unlike single-walled carbon nanotubes show diffraction peak around 25° ((002) diffraction → interlayer spacing (3.4 Å))^45^, (calculated according to the Bragg-equation assuming λ = 1.5418 Å (Cu K_α_ X-rays). which is at significantly smaller *d*-spacing than for rGO-PNEAM (= > d = 4.62 Å), as unlike MWCNT, rGO-PNEAM also contained the surface polymerized PNEAM, which must be present between the two graphene sheets. This could make this composite an interesting material to study the behavior of polymers in 2D-confinement.

Alternatively, the peak might be caused by 2D-crystalline PNEAM. It is known that some polyacrylamides can crystallize, while PNEAM and PNIPAM cannot under normal circumstances. However, the crystallization in confinement is physically very different^46^. DSC-scans did not show any sign of crystallization. However, considering the high T_g_ of NIPAM and NEAM (ca. 140 °C) and the low decomposition temperature of these polymers ~ 400 °C, speculatively, their T_m_ would be too close to the decomposition temperature.

The other interesting question arising from that finding is why PNEAM causes the rGO to “roll up”, while PNIPAM and NEAM-NIPAM copolymers don’t. The chemical difference between NIPAM and NEAM is only one additional methyl group, which increases sterical hindrance and hydrophobicity in PNIPAM (→LCST). The copolymers cannot form these tubular structures, as the polymer chains’ local potential is monomer sequence dependent and, thus, inherently heterogeneous.

Hence, we conclude that this peak is caused by the rolled up graphene sheets facilitated by PNEAM, but further investigations have to elucidate what physical mechanism is.

## Conclusions

Composites of graphene oxide and RAFT-agents were synthesized by click-chemistry. During click-reactions, GO was also reduced by ascorbic acid. The resulting nanohybrids are water-dispersible nanoparticles, which have a hydrophobic rGO-core. This allows for including them into water-based systems, e.g. for making electronics for aqueous environments.

The thermoresponsiveness (LCST in water) was tuned in the very broad temperature range between 33 and 70 °C, following an almost linear dependence with monomer ratio, strongly suggesting a statistical copolymer, in which comonomers possess a not too different interaction pattern with water, due to chemical similarity of NEAM and NIPAM.

Considering this, however, it was surprising to find significant differences on microscopic scale. SEM and AFM showed hollow rod-like structures for rGO-PNEAM. XRD showed a small peak, corresponding to d-spacing ≈4.6 Å. Based on previous reports of MWCNT, this XRD-peak could correspond to a distorted graphene layer stacking peak or, alternatively, to PNEAM crystallizing under graphene sheets’ confinement and/or nucleation effect.

In the future, we hope to elucidate this interesting finding found for rGO-PNEAM and whether it can also occur for other polymers.

## Methods

### Materials

Graphite was purchased from Alpha Aesar (200 μm). NIPAM (Macklin, 98%) was recrystallized from n-hexane before use. NEAM (Sigma Aldrich, 99%) was purified by passing through an alumina column. All the other chemicals and solvents were purchased from Shanghai Macklin Biochemical Co., Ltd., were of analytical grade and were used without further purification.

### Preparation of RAFT-CTA modified graphene

Graphite oxide was prepared by modified Hummers’ method^47^ and functionalized with azide with reaction of GO with sodium azide in DMF for 48 h to obtain GO-N_3_^48^. S-1-dodecyl-S’-(α, α ‘-dimethyl- α”-acetic acid) trithiocarbonate was functionalized with propargyl alcohol in the presence of EDC and DMAP in dichloromethane according to a previously reported method in literature^49^ (alkyne-RAFT). 500 mg of GO-N_3_ was exfoliated in 250 mL DMF by using tip sonication to obtain a dispersion with a solid content of 2 mg mL^−1^. RAFT-CTA (300 mg, 0.71 mmol), CuSO_4_.5H_2_O (100 mg, 0.4 mmol) and sodium ascorbate (200 mg, I mmol) was added to the suspension and stirred for 48 h at room temperature. The reaction vessel was sonicated 2 times per day. It was finally washed with DMF, water and EtOH several times and dried in vacuum overnight. In order to determine the sulfur content and, subsequently, the amount of the RAFT-CTA grafted to the rGO sheets elemental analysis, as well as EDS, was used. The result shows that the sulfur content is 2.29 wt.%, so the RAFT-CTA in the nanocomposite is about 0.24 mmol g^−1^ (C 87.14 wt.%, O 10.54 wt.%, S 2.29 wt.%) which is close to the number by EDS (2.32 wt.%).

### Preparation of polymer nanohybrids

rGO-polymer nanohybrids were prepared by RAFT-technique in DMF at 60 °C in presence 2,2′-azobis(isobutyronitrile) (AIBN) with different compositions of monomers. NIPAM and NEAM were used as monomers in pure form and also in ratios of 1:3, 1:1, and 3:1. 100 mg rGO-RAFT agent was taken in 8 mL DMF in each vial and ultra-sonicated for 30 min to make it completely disperse followed by purging with nitrogen for 30 min. The monomers (NEAM and NIPAM) were also purged with nitrogen separately for 30 min. In the Schlenk tubes the required amount of monomer and AIBN were taken, vacuum was created and then filled with nitrogen using Schlenk system. This process was repeated for three times. Finally, the nitrogen purged DMF and monomers were added to the reaction mixture using a degassed syringe and polymerizations were performed at 60 °C under nitrogen atmosphere for desired time. The same procedure was for the copolymers by adding mixture of monomers at different ratios. After the polymerization was done, the reactions were quenched by freezing in liquid nitrogen. The polymer nanocomposites were separated by centrifugation. The free polymers (sacrificed polymer from nanocomposites) were purified by precipitation from diethyl ether and dried under vacuum. The number average molar mass M_n_ and polydispersity of the polymers in nanocomposites were determined by size exclusion chromatography (SEC) of the free polymers in THF at 40 °C with flow rate of 1 mL min^−1^.

### Characterization

The Fourier-transform infrared spectrum (FTIR) was conducted on a Nicolet 6700 (Thermo Scientific, USA). The samples were sputtered with gold and examined using thermal field emission scanning electron microscope (SEM; Hitachi, SU-70) at an activation voltage of 15.0 kV. Raman spectra were recorded from 500 to 3000 cm^−1^ on a inVia confocal Raman microscope (Renishaw, Great Britain) while X-ray diffraction (XRD) was measured on Brucker D8 Advanced (Germany). The weight loss upon heating analysis was performed on a TA Q50 in a nitrogen atmosphere at a heating rate of 10 °C min^−1^ from RT to 700 °C. AFM measurements of the nanohybrids were done by using a AFM Dimension Icon (Bruker, USA) in Force Volume (FV) mechanical imaging mode. The GO sample was prepared by deposition of a GO dispersion in water (0.01 mg mL^−1^) while the nanocomposite samples were prepared by spin coating (3000 rpm for 3 min) the dispersion in water onto a silicon wafer, respectively and dried under N_2_ flow. Indentation experiments were performed using sharp pyramidal (17° average half-angle aperture) probes, SCANASYST-AIR (Bruker, USA) with elastic constant k = 0.39 N/m and nominal apex radius 5 nm. Considering indentation depth up to 40 nm and the geometry of the indenter, Sneddon model was preferred for Young’s modulus calculation^50^. The rheological experiments of the LCST were carried out on an Anton Paar MCR 302 rheometer using a 25 mm cone geometry and a solvent trap to maintain a solvent saturated atmosphere. A heating rate of 2 K/min at a deformation γ_0_ of 10% and a frequency ω of 10 rad s^−1^ was used to ensure that the temperature in the sample is homogeneous and to avoid a significant difference between sensor and sample temperature as well as mechanical deformations in the linear range of deformation. In this setup, G’ becomes unreliably detectable below 0.01 Pa (quantitatively). Qualitatively, the data become unreliable below ca. 10–4 Pa. Furthermore, the LCST leads to a phase separation, which goes from microscopic to macroscopic phase separation as temperature above the LCST increases. As soon as the phase separation becomes too severe, a decrease in G’ is observed, as the polymers in the sample start detaching from the plates and, hence, G’ decreases. Obviously these data cannot be considered to be reliable anymore and were therefore cropped.

## Additional Information

**How to cite this article:** Namvari, M. *et al*. Reduced graphene oxide composites with water soluble copolymers having tailored lower critical solution temperatures and unique tube-like structure. *Sci. Rep.* **7**, 44508; doi: 10.1038/srep44508 (2017).

**Publisher's note:** Springer Nature remains neutral with regard to jurisdictional claims in published maps and institutional affiliations.

## Supplementary Material

Supporting Information

## Figures and Tables

**Table 1:** Summary of obtained polymer data.

	PNIPAM (mM)	PNEAM (mM)	Conversion^a^ (%)	M_n_ (g/mol)×10^3^	PDI (M_w_/M_n_)	LCST (°C)
*rGO-PNIPAM*	12	0	85	6.77	1.31	30.0
*rGO-PNIAPM:PNEAM 3:1*	9	3	77	3.98	1.50	43.3
*rGO-PNIAPM:PNEAM 1:1*	6	6	75	3.26	1.51	52.4
*rGO-PNIAPM:PNEAM 1:3*	3	9	76	2.02	1.45	60.9
*rGO-PNEAM*	0	12	80	1.04	1.34	68.5

^a^determined gravimetrically.
