# Supplementary material for: Reduced graphene oxide composites with water soluble copolymers having tailored lower critical solution temperatures and unique tube-like structure
Source: Sci Rep. 2017 Mar 14;7:44508. doi: 10.1038/srep44508 (PMC5349575; doi:10.1038/srep44508)
Supplement: Supporting Information [file srep44508-s1.pdf]

Reduced graphene oxide composites with water soluble copolymers having tailored lower critical solution temperatures and unique tube-like structure

*Mina Namvari[1,2], Chandra S. Biswas[1,2], Massimiliano Galluzzi[1,2], Qiao Wang[1], Bing Du[1], Florian J. Stadler[1]**

[1]College of Materials Science and Engineering, Shenzhen Key Laboratory of Polymer Science and Technology, Guangdong Research Center for Interfacial Engineering of Functional Materials, Nanshan District Key Lab for Biopolymers and Safety Evaluation, Shenzhen University, Shenzhen 518060, PR China

[2]Key Laboratory of Optoelectronic Devices and System of Ministry of Education and Guangdong Province, College of Optoelectronic Engineering, Shenzhen University, Shenzhen, People's Republic of China

* Prof. Florian J. Stadler

+86-0755-26538236

fjstadler@szu.edu.cn

Results and discussion

FT-IR spectroscopy provides information about the structures of the materials, as shown in Fig. SI1. The FT-IR spectra of GO clearly showed the characteristic absorption peaks at 1091, 1392, 1631, 1735, and 3332 cm^{-1}, corresponding to the stretching modes of C-O-C, C-OH, C=C, C=O and O-H, respectively. For $GO-N_3$, peak at 2054 cm^{-1} was observed and could be assigned to the absorption of the azide groups while alkyne-RAFT shows a weak peak at 2120 cm^{-1} attributed to alkyne group, and trithioester group present a peak at 1090 cm^{-1}. After clicking $GO-N_3$ and alkyne-RAFT together, the peaks related to azide and alkyne groups disappeared and stretching bands of alkyl group on the RAFT chain were observed in the spectrum. In addition, the intensity of C-O and O–H peaks were significantly reduced, indicating that during click reaction, the GO sheets have been reduced as well.[1] This is an added advantage for click-coupling reactions to obtain graphene sheets without the need for a further reduction step. In the polymer nanohybrid spectra, the typical absorption peaks of polyacrylamides include an absorption around 1550 cm^{-1} due to N-H bending, 1600 cm^{-1} C=O and 3500 cm^{-1} N-H stretching.[2]

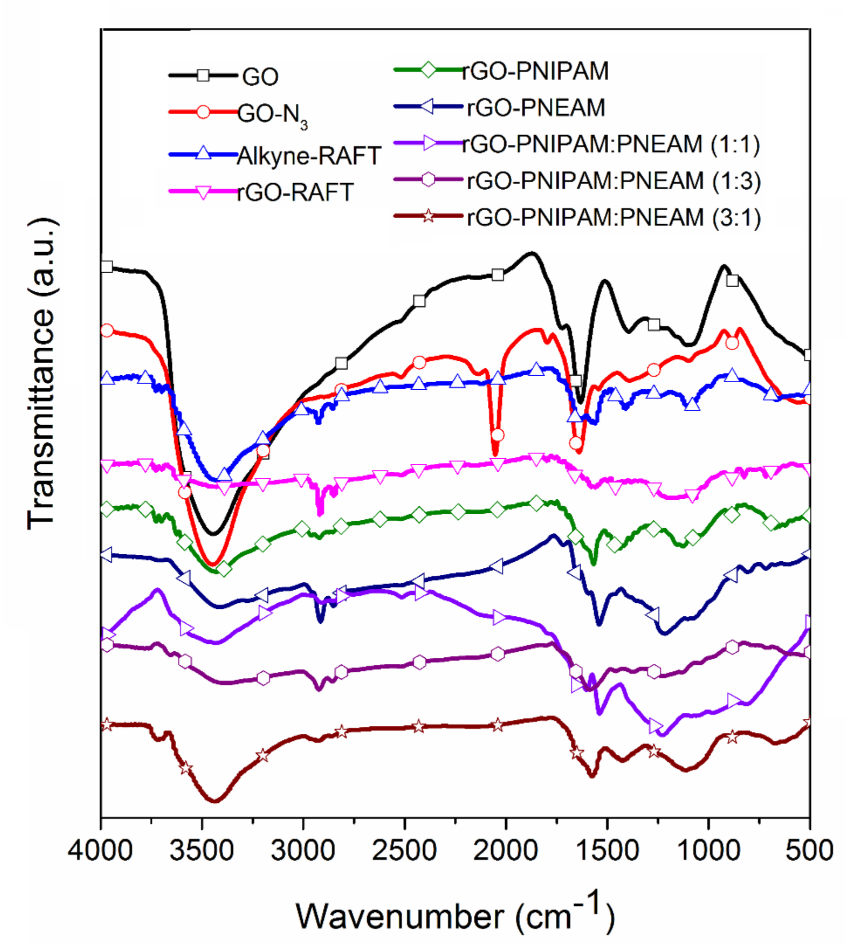

Fig. SI1. FTIR and (B) Raman spectra of GO, GO-N_3, alkyne-RAFT, rGO-RAFT, and polymer nanohybrids.

Fig. SI2 shows Raman data for GO, GO-N_3, rGO-RAFT, rGO-PNIPAM, and rGO-PNEAM. Graphite shows only one peak (G band), which is related to sp^2 hybridization in a graphitic two dimensional hexagonal lattice, while GO showed another peak named D band at 1360 cm^{-1} ascribed to sp^3 hybridization (C atoms of defects and disorder) besides the G band that was observed at 1586 cm^{-1}. In graphitic materials functionalization leads to a blue shift in G band and a red shift in D band. Shift in G band to higher wavenumbers could be due to the influence of defects and isolated double bonds produced by the reduction process of GO. However, D band still existed after functionalization since it was difficult to restore GO completely into graphene even after reduction.[3,4] The D bands displayed by homopolymer nanohybrids were detected at 1351 and 1354 cm^{-1} and for copolymer ones were observed at 1341, 1345, and 1342 cm^{-1} a relatively lower value when compared to that displayed by GO (1360 cm^{-1}), indicating the chemical modification of GO surface or electron transfer between the polymers and GO. I_D/I_G is used to get information about the disordered and ordered domains of graphene. Grafting has led to an increase in I_D/I_G in the following order: rGO-PNIPAM > rGO-copolymer 1:1 > rGO-PNEAM = rGO-copolymer 3:1 > rGO-copolymer 1:3 > GO-N_3 > rGO-RAFT >

GO (Table SI1). The increased I_D/I_G ratio reflects decreased average size of the sp^2 domains in the related nanocomposites.[5]

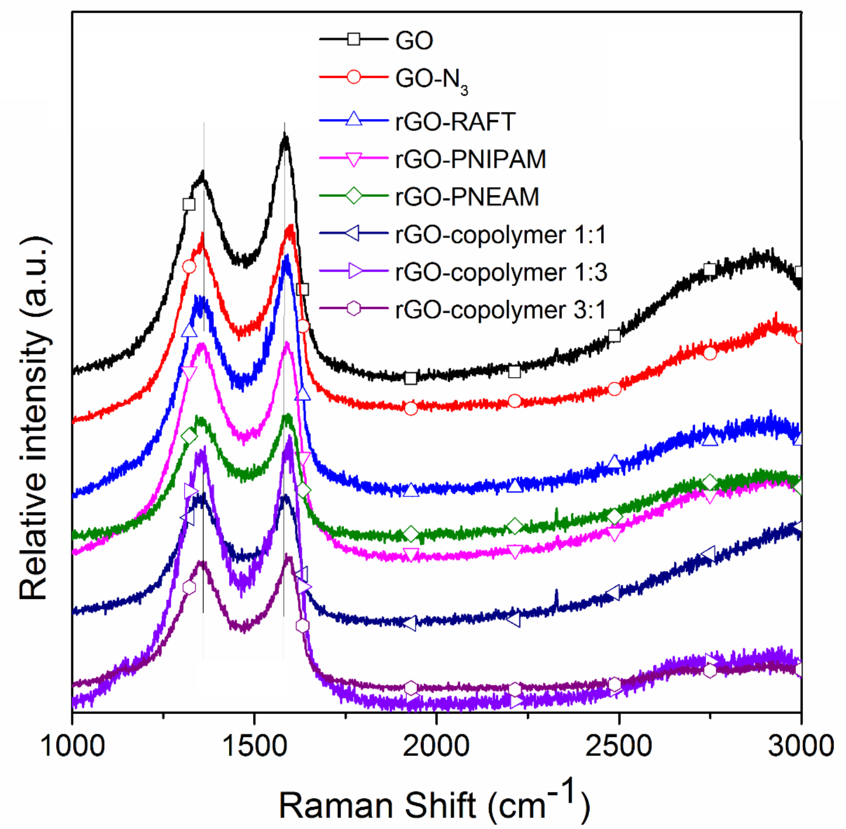

Fig. SI2. Raman spectra of GO, GO-N$_3$, alkyne-RAFT, rGO-RAFT, and polymer nanohybrids.

Table SI1. Chemical shifts of D and G band and I_D/I_G of GO, GO-N$_3$, alkyne-RAFT, rGO-RAFT, and polymer nanohybrids.

Material	**D band (cm^{-1})**	**G band (cm^{-1})**	**I_D/I_G**
GO	1360	1586	0.85
GO-N$_3$	1354	1597	0.94
rGO-RAFT	1347	1589	0.92
rGO-PNIPAM	1351	1587	1.01
rGO-PNEAM	1354	1582	0.98
rGO-copolymer 1:1	1341	1591	1.00
rGO-copolymer 1:3	1345	1610	0.96
rGO-copolymer 3:1	1342	1615	0.98

The zeta potential, average size and conductivity of the polymer nanocomposites were measured at 25°C (Fig. SI3). The Z-average for GO, rGO-PNIPAM, rGO-PNEAM, and rGO-copolymer 1:1 were 437, 1300, 1004, 1339 nm, while PDI was 0.477, 0.653, 0.761, and 0.741, respectively. The increase in size of the nanomaterials were due to the introduction of polymers on the graphene sheets. Even though the size of the graphene sheets reduced in each step of functionalization, the increase in the average size of the nanocomposites could be attributed to this fact that water-soluble polymers stretch out in water.

This is also the reason why the polydispersities are relatively high. Depending on the angle under which laser checks the nanohybrids, their size varies, which causes a broadening of the size distribution.

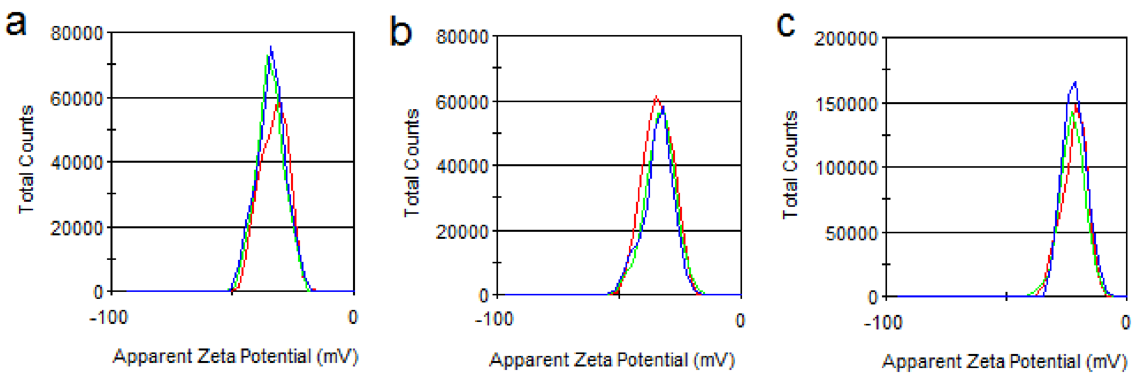

Fig. SI3. Zeta potential of (a) rGO-PNIPAM, (b) rGO-PNEAM, (c) rGO-copolymer 1:1 at 25°C.

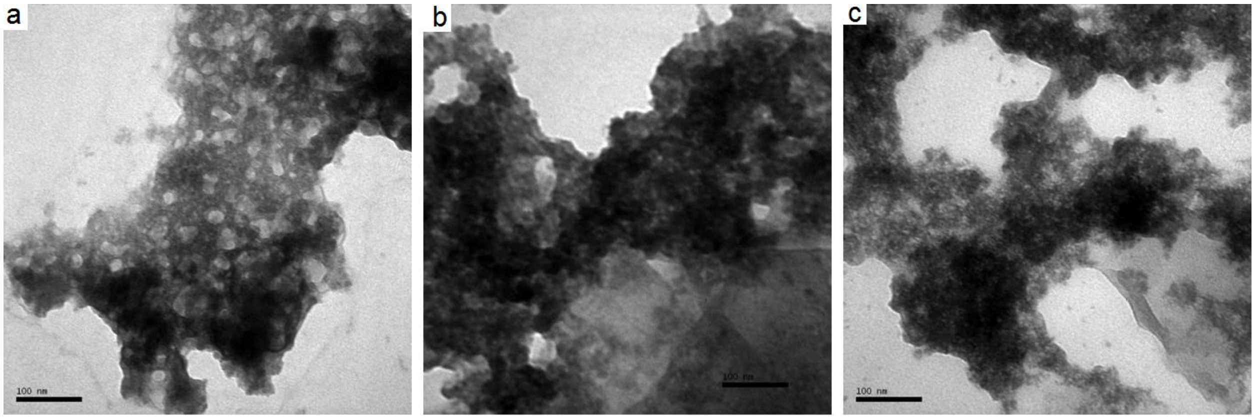

Fig. SI4. TEM images of (a) rGO-PNIPAM, (b) rGO-PNEAM, and (c) rGO-copolymer 1:1.

An estimation of the grafting density of the can be done when combining several geometric considerations.

1. Elemental analysis found ca. 0.24 mmol RAFT-agent per gram of RGO-RAFT composite. Considering the molar mass of 448 g/mol of the RAFT-agent including the coupling groups, we can conclude that the composite contains ca. 89.3 wt. % RGO and 10.7 wt. % RAFT-agents.

2. Bonaccorso et al. [6] reported the theoretical specific surface area (SSA) to be 2630 m^2/g. Considering the graphene content, this translates into ca. 2347 m^2/g for the RGO-RAFT composite. This assumes that the influence of the oxygen and of the sheet edges is negligible.

3. Based on these numbers and the Avogadro-number, the grafting density can be calculated to be 6.37×10^{16} RAFT-molecules per m^2, which translates into 15.7 nm^2 per grafted RAFT-molecule. This number includes both sides of the RGO-sheet.

4. When assuming that the 15.7 nm^2 encompass a circular area, the radius would be 2.23 nm. The length of the RAFT-agent including the coupling group is 2.7 nm (assuming a stretched molecule) and, thus, on approximately the same length scale. It is clear that this assumption is not 100% correct, as – when assuming an ideal hexagonal packing of the grafted groups – the area should be hexagonal, but the grafting does not follow an ordered but rather a statistical packing and consequently, we can only calculate average areas per RAFT-agent. For the purpose of this estimation, the assumption of circular areas of influence is sufficient for getting an idea of the grafting density.

Considering that based on these assumptions 2 RAFT-molecules are ca. 2 radii (=4.47 nm) separated, while the RAFT-agents' length is somewhat less than that, it becomes clear that this grafting density automatically leads to a higher surface roughness.

References

1 Namvari, M. & Namazi, H. Clicking graphene oxide and Fe3O4 nanoparticles together: an efficient adsorbent to remove dyes from aqueous solutions. *International Journal of Environmental Science and Technology* **11**, 1527-1536, doi:10.1007/s13762-014-0595-y (2014).

2 Yang, Y. F. *et al.* Synthesis of PNIPAM Polymer Brushes on Reduced Graphene Oxide Based on Click Chemistry and RAFT Polymerization. *Journal of Polymer Science Part a-Polymer Chemistry* **50**, 329-337, doi:10.1002/pola.25036 (2012).

3 Liu, Z. Z. *et al.* Preparation of graphene/poly(2-hydroxyethyl acrylate) nanohybrid materials via an ambient temperature "grafting-from" strategy. *Polymer Chemistry* **6**, 311-321, doi:10.1039/c4py00903g (2015).

4 Liu, Z., Lu, G., Li, Y., Li, Y. & Huang, X. Click synthesis of graphene/poly(N-(2-hydroxypropyl) methacrylamide) nanocomposite via "grafting-onto" strategy at ambient temperature. *RSC Advances* **4**, 60920-60928, doi:10.1039/C4RA07825J (2014).

5 Li, B. *et al.* Tunable Functionalization of Graphene Oxide Sheets through Surface-Initiated Cationic Polymerization. *Macromolecules* **48**, 994-1001, doi:10.1021/ma5026237 (2015).

6 Bonaccorso, F. *et al.* Graphene, related two-dimensional crystals, and hybrid systems for energy conversion and storage. *Science* **347**, doi:10.1126/science.1246501 (2015).
